# Potentiation of isokinetic torque is velocity-dependent following an isometric conditioning contraction

**DOI:** 10.1186/2193-1801-2-554

**Published:** 2013-10-22

**Authors:** Atsuki Fukutani, Naokazu Miyamoto, Hiroaki Kanehisa, Toshimasa Yanai, Yasuo Kawakami

**Affiliations:** Research Organization of Science and Technology, Ritsumeikan University, 1-1-1 Noji-higashi, Kusatsu, Shiga, 525-8577 Japan; Japan Society for the Promotion of Science, 5-3-1 kojimachi, Chiyoda-ku, Tokyo, 102-8472 Japan; National Institute of Fitness and Sports in Kanoya, Shiromizu-cho, Kagoshima, Kagoshima, 891-2393 Japan; Faculty of Sport Sciences, Waseda University, 2-579-15 Mikajima, Tokorozawa, Saitama, 359-1192 Japan

**Keywords:** Postactivation potentiation, Conditioning contraction, Myosin regulatory light chain, Twitch contraction, Plantar flexion, Electromyography

## Abstract

Not only twitch torque but also the maximal voluntary concentric torque increases after a high-intensity contraction (conditioning contraction). The purpose of this study was to test the hypothesis that the increase in the maximal voluntary concentric torque induced by a conditioning contraction is prominent when tested at fast angular velocities conditions. Twelve healthy male participants performed the maximal voluntary isometric plantar flexion for six seconds as a conditioning contraction. Before and after the conditioning contraction, peak torques during the maximal voluntary concentric plantar flexions were measured at 30°/s (slow) and 180°/s (fast), each of which was carried out in a separate condition. Isometric twitch torque was also recorded before and after the conditioning contraction in each of the two velocity conditions to confirm the extent of the positive effect of the conditioning contraction. The extent of increase in isometric twitch torque was similar between the two velocity conditions, whereas the maximal voluntary concentric torque increased significantly only in the fast velocity condition (*p* = 0.003). These results support the hypothesis and indicate that the maximal voluntary concentric torque can be potentiated by the conditioning contraction if the joint angular velocity during the maximal voluntary concentric contraction is sufficiently high.

**Electronic supplementary material:**

The online version of this article (doi:10.1186/2193-1801-2-554) contains supplementary material, which is available to authorized users.

## Introduction

The twitch force generated by isolated muscle fibers increases shortly after a high-intensity contraction of the same muscle (MacIntosh et al. 2008); (Manning and Stull 1982). This phenomenon is called posttetanic potentiation (Abbate et al. 2000). A similar phenomenon is observed also in vivo, where the twitch joint torque elicited after a high-intensity voluntary contraction increases (postactivation potentiation [PAP] (Ebben 2006); (Sale 2002); (Vandervoort et al. 1983). The preceding voluntary contraction for inducing PAP is referred to as a conditioning contraction (Tillin and Bishop 2009). The underlying mechanism of the increase in twitch torque is considered to be the phosphorylation of the myosin regulatory light chain, which leads an increase in the number of the attached cross bridges at a given Ca^2+^ concentration released from the sarcoplasmic reticulum (Kamm and Stull 2011); (Rassier and MacIntosh 2000); (Sweeney et al. 1993).

It has been under debate whether the conditioning contraction increases the force or torque generated by high-intensity contractions. Some studies reported that the electrically-evoked maximal isometric force was not increased by a conditioning contraction (Vandenboom et al. 1993;1995;1997) whereas others observed increases in the maximal voluntary concentric torque (Fukutani et al. 2012); (Miyamoto et al. 2011). One of the possible reasons for this discrepancy is that the type of contraction (i.e., isometric or concentric contractions) used to test the effect of the conditioning contraction varied among these studies. In the previous studies reporting positive potentiation effects, concentric contractions at relatively fast joint angular velocities (180°/s in plantar flexion) were used for the testing (Fukutani et al. 2012); (Miyamoto et al. 2011). Babault et al. (2008) reported that the extent of increase in twitch torque induced by the conditioning contraction increased when the joint angular velocity during a twitch contraction increased, which indicates that the sensitivity to the positive effect of a conditioning contraction is high when the joint angular velocity is high. The extent of increase in the force induced by the conditioning contraction becomes smaller as the contraction intensity for examining the effect of the conditioning contraction increases (MacIntosh and Willis 2000). Thus, the torque attained during a high-intensity contraction may increases when the sensitivity to the positive effect of conditioning contraction is sufficiently high at, or higher than, a certain joint angular velocity. If so, there is possibility that if a lower joint angular velocity was used than the one used in previous studies (180°/s, Fukutani et al. 2012 and Miyamoto et al. 2011), the increase in the maximal voluntary concentric torque may have been small or negligible. In this study, therefore, we investigated the effects of a conditioning contraction on the increase in the maximal voluntary concentric torque at not only fast (180°/s) but also slow (30°/s) joint angular velocities. We hypothesized that the maximal voluntary concentric torque increases only in the fast joint angular velocity condition.

## Methods

### Participants

Twelve young adult men (age: 25.0 ± 2.3 years, height: 1.73 ± 0.05 m, body mass: 65.7 ± 5.9 kg) participated in this study. Each participant was a healthy undergraduate or graduate student who had not participated in a regular resistance training program. Each participant received explanation about the procedures of the experiment before voluntarily participating in this study. This study was approved by the Ethics Committee on Human Research of Waseda University. During the day of experiment, the participants did not take caffeine that is known to affect the extent of PAP (MacIntosh and Gardiner 1987).

### Experimental setup

Plantar flexors were selected as the target muscles in this experiment because previous studies that reported an increase in the maximal voluntary concentric torque selected plantar flexors as the target muscles (Fukutani et al. 2012); (Miyamoto et al. 2011). Each participant lay on an isokinetic dynamometer (CON-TREX, CMV AG, Switzerland) with the right knee and hip joints fully extended. The right foot was tightly secured to the dynamometer’s footplate, and the right thigh was fixed to the dynamometer’s bench with a non-elastic strap to minimize unwanted movements. The center of rotation of the footplate and center of the ankle joint were aligned visually. During the twitch and conditioning contractions, the ankle joint angle was set at 0° (anatomical position). The joint angular velocity of the maximal voluntary concentric contraction was set at 30°/s and 180°/s (slow and fast conditions, respectively). The range of motion of the maximal voluntary concentric contraction was set from -15° (dorsiflexion) to 30° (plantar flexion) in the fast velocity condition and from -15° to -5° in the slow velocity condition to minimize the difference of the contraction duration between the two velocity conditions. In a preliminary study, we confirmed that peak torque during the maximal voluntary concentric contraction in each velocity condition was recorded within these ranges of motion. Subjects were instructed to perform the fast and slow maximal voluntary concentric contraction as forcibly as possible.

Surface electromyography (EMG) signals were obtained from the medial gastrocnemius (MG), lateral gastrocnemius (LG), soleus (SOL), and tibialis anterior (TA). After shaving, abrasion and cleaning with the alcohol, we placed the pre-amplified bipolar differential electrodes (Ag/AgCl, input impedance: 1 MΩ, electrode bar size: 1 × 10 mm each, DE-2.1, DELSYS, USA) on the surface of each muscle with an inter-electrode distance of 10 mm (CMRR: 92 dB, Gain: 1000, Band-pass filter: 20–450 Hz, Bagnoli 8 EMG System, DELSYS, Boston, MA, USA). The reference electrode was placed over the left lateral malleolus (11 mm diameter, Blue sensor, N-00-S, Ambu, Denmark). The analog data of EMG signals, torques and joint angles were converted into the digital data using a 16-bit analog-to-digital converter (Power-lab/16SP, ADInstrument, Australia). The sampling frequency was set at 4 kHz.

The posterior tibial nerve was stimulated percutaneously to evoke an isometric twitch contraction of the plantar flexor muscles. A cathode (11 mm diameter, Blue sensor, N-00-S, Ambu, Denmark) was placed over the popliteal fossa, and an anode (40 × 50 mm, VIASYS, Healthcare, USA) was placed over the ventral aspect of the thigh near the patella. Single rectangular pulses of 500 μs duration were delivered from a high-voltage stimulator (SEN-3301, Nihon Kohden, Japan) with a specially modified isolator (SS-1963, Nihon Kohden, Japan). The stimulus intensity was determined prior to the experiment by increasing the voltage until the corresponding torque reached a plateau with joint angle at 0°. The stimulus intensity was set at 20% above the intensity, which no further increase in twitch torque was confirmed. The stimulation intensity ranged from 40 to 90 volts.

### Protocol

**Figure 1:**
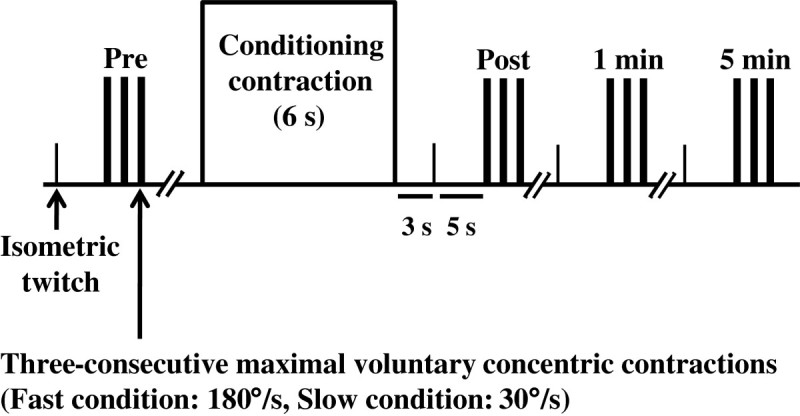
**Experimental protocol.** Pre: Before the conditioning contraction; Post: Immediately after the conditioning contraction.

First, an isometric twitch contraction of the plantar flexors was elicited and the peak torque value was recorded as the isometric twitch torque with no potentiation. This procedure was repeated two times to confirm the reproducibility. Next, the participant performed a familiarization task before the main experiment. The familiarization task consisted of several voluntary concentric contractions (more than three times in each condition) of the plantar flexors at 30°/s and 180°/s with submaximal and maximal effort. After the completion of the familiarization task, the participant rested for more than 10 minutes (Baudry et al. 2008) to avoid the effect of PAP caused by the familiarization task on the main experiment. The protocol of the main experiment is shown in Figure 1. First, an isometric twitch contraction was elicited in a similar fashion for the two velocity conditions to calculate the extent of increase in isometric twitch torque as an index of the positive effect of the conditioning contraction. After that, the participant was asked to perform three-consecutive maximal voluntary concentric contractions of the plantar flexors. To avoid the effect of PAP induced by three-consecutive maximal voluntary concentric contractions on the outcomes of the following trials, a rest period of approximately five minutes was allowed after the three-consecutive maximal voluntary concentric contractions. After confirming that the difference of the isometric twitch torque was within ±10% deviation and within ±2 Nm compared to that with no potentiation, the participant performed the maximal voluntary isometric contraction of the plantar flexors for six seconds as a conditioning contraction. Immediately after the conditioning contraction (approximately three seconds later), an isometric twitch contraction was elicited and then (approximately five seconds later), three-consecutive maximal voluntary concentric contractions were performed at 30°/s or 180°/s. In addition, the isometric twitch contraction and the three-consecutive maximal voluntary concentric contractions were conducted one and five minutes after the conditioning contraction. Each velocity condition was separated by a rest period of more than 10 minutes to avoid the effect of PAP caused by the conditioning contraction (Vandervoort et al. 1983) and three-consecutive maximal voluntary concentric contractions which were performed immediately, and 1 and 5 min after the conditioning contraction on the next velocity condition. The second condition was performed after confirming that the difference of the isometric twitch torque was within ±10% deviation compared to that with no potentiation. The order of the two velocity conditions was randomized.

### Measurements

**Figure 2:**
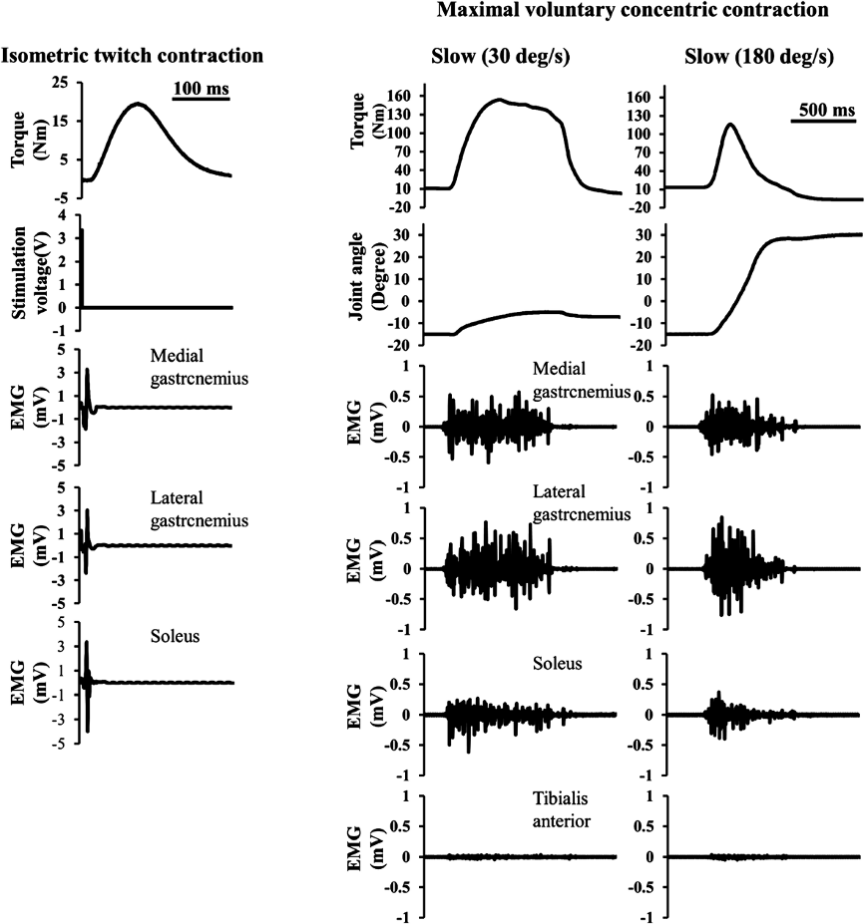
**Typical waveforms of torque and EMG obtained during isometric twitch and the maximal voluntary concentric contractions.** Left: Raw data recorded during isometric twitch contraction, Right: Raw data recorded during the maximal voluntary concentric contractions in slow and fast conditions.

Typical waveforms of torque and EMG obtained during isometric twitch and the maximal voluntary concentric contractions are shown in Figure 2. Peak torque and the peak-to-peak amplitude of the M-wave during the isometric twitch contraction were adopted as the isometric twitch torque and M-wave amplitude, respectively. The contraction in which the highest peak torque was attained among the three-consecutive maximal voluntary concentric contractions was adopted for subsequent analyses. The peak torque attained during the maximal voluntary concentric contraction was recorded as the maximal voluntary concentric torque. The root-mean-square value of the EMG signal (RMS_EMG_) of each muscle was calculated over 200 ms period in the middle of the maximal voluntary concentric contraction (from 100 ms before to 100 ms after the instance of peak torque). A 20-Hz high-pass filter was used to minimize the effect of motion artifact on the RMS_EMG_ value (De luca et al. 2010). Each parameter recorded at three time points (i.e., immediately, 1 and 5 minutes after the conditioning contraction) was expressed as the value relative to that recorded before the conditioning contraction (%change). The RMS_EMG_ normalized by the M-wave was also calculated as an index of central fatigue. Furthermore, the ankle joint angle at peak torque during the maximal voluntary concentric contraction occurred was measured.

Mean value of torque and RMS_EMG_ of plantar flexors during the conditioning contraction with a 500 ms duration that torque signal was stable were calculated in the first and second conditions to confirm whether subjects exerted conditioning contraction with the same intensity in first and second conditions.

We confirmed the reproducibility of the twitch torque between the two trials conducted at the beginning of the experiment and the maximal voluntary concentric torque among three-consecutive trials performed before the conditioning contraction in each velocity condition. For the twitch torque, the coefficient of variance was 1.5 ± 1.2% and the intra-class correlation was 0.99. For the maximal voluntary concentric torque, the coefficient of variance was 5.6 ± 2.2% at the 180°/s condition and 3.8 ± 2.6% at the 30°/s condition. The intra-class correlation was 0.78 at the 180°/s condition and 0.92 at the 30°/s condition.

### Statistics

Descriptive data are presented as mean ± SD. The isometric twitch torque, M-wave amplitude, maximal voluntary concentric torque, RMS_EMG_ during the maximal voluntary concentric contraction, the values of RMS_EMG_ normalized by the M-wave and ankle joint angle at peak torque during the maximal voluntary concentric contraction occurred were tested by repeated-measures two-way ANOVA (velocity × time point). If the interaction was significant, additional repeated-measures one-way ANOVAs with a subsequent post-hoc test with Bonferroni’s correction was used for examining the time-course changes in each velocity condition. In addition, paired *t*-test was used for examining the difference between the two velocity conditions at each time point. Mean value of torque and RMS_EMG_ of plantar flexors during the conditioning contraction was compared by paired *t*-test. The level of significance was set at *p* < 0.05. A software package (IBM SPSS Statistics 20.0, IBM, USA) was used for the statistical analyses.

## Results

Absolute values of torque and EMG obtained during twitch contraction and the maximal voluntary concentric contraction are shown in Table 1.

**Table 1:** **Absolute value of torque and EMG during isometric twitch contraction and the maximal voluntary concentric contraction**

Absolute values of torque and EMG				
Isometric twitch torque (Nm)	Pre	Post	1 min	5 min
Fast	24.1 ± 4.5	39.2 ± 5.1	31.4 ± 5.4	26.7 ± 5.0
Slow	23.4 ± 3.9	39.0 ± 5.5	31.8 ± 5.5	26.0 ± 4.8
Maximal voluntary concentric torque (Nm)	Pre	Post	1 min	5 min
Fast	94.6 ± 12.3	100.7 ± 13.4	99.0 ± 16.0	97.2 ± 12.3
Slow	149.0 ± 24.5	149.1 ± 22.7	147.2 ± 22.9	149.4 ± 19.9
M-wave of medial gastrcnemius (mV)	Pre	Post	1 min	5 min
Fast	3.2 ± 2.1	3.0 ± 2.0	3.3 ± 2.1	3.4 ± 2.1
Slow	3.3 ± 2.1	3.1 ± 2.0	3.3 ± 2.1	3.4 ± 2.1
M-wave of lateral gastrcnemius (mV)	Pre	Post	1 min	5 min
Fast	4.3 ± 1.7	4.4 ± 1.6	4.6 ± 1.9	4.5 ± 1.8
Slow	4.3 ± 1.7	4.4 ± 1.6	4.6 ± 1.7	4.6 ± 1.8
M-wave of soleus (mV)	Pre	Post	1 min	5 min
Fast	3.7 ± 2.1	4.0 ± 2.0	3.8 ± 2.1	3.7 ± 2.2
Slow	3.9 ± 2.1	4.2 ± 1.9	4.0 ± 2.1	3.9 ± 2.1
RMS_EMG_ of medial gastrocnemius (mV)	Pre	Post	1 min	5 min
Fast	0.28 ± 0.11	0.26 ± 0.07	0.26 ± 0.10	0.25 ± 0.07
Slow	0.25 ± 0.08	0.24 ± 0.06	0.22 ± 0.05	0.23 ± 0.05
RMS_EMG_ of lateral gastrocnemius (mV)	Pre	Post	1 min	5 min
Fast	0.28 ± 0.09	0.26 ± 0.10	0.25 ± 0.10	0.26 ± 0.11
Slow	0.24 ± 0.07	0.23 ± 0.09	0.21 ± 0.08	0.23 ± 0.11
RMS_EMG_ of soleus (mV)	Pre	Post	1 min	5 min
Fast	0.16 ± 0.05	0.16 ± 0.06	0.16 ± 0.07	0.16 ± 0.06
Slow	0.16 ± 0.05	0.14 ± 0.04	0.14 ± 0.06	0.15 ± 0.04
RMS_EMG_ of tibialis anterior (mV)	Pre	Post	1 min	5 min
Fast	0.04 ± 0.01	0.04 ± 0.01	0.04 ± 0.01	0.04 ± 0.01
Slow	0.03 ± 0.01	0.03 ± 0.01	0.03 ± 0.01	0.03 ± 0.01

Data are shown as means ± SDs. Pre: Before the conditioning contraction; Post: Immediately after the conditioning contraction.

**Figure 3:**
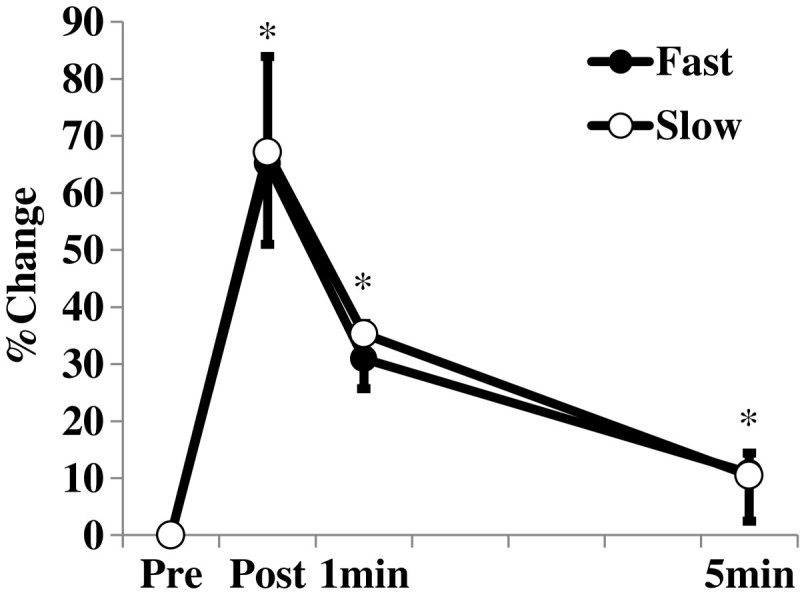
**Relative changes in isometric twitch torque.** Relative changes in isometric twitch torque after the conditioning contraction. Pre: Before the conditioning contraction; Post: Immediately after the conditioning contraction. *: Significantly different (*p* < 0.05) from Pre values in both conditions.

For the isometric twitch torque, there was a significant main effect of time point (*F* value = 138.874, *p* < 0.001) without the interaction of time point and velocity (*F* value = 2.623, *p* = 0.067) (Figure 3). Post-hoc tests revealed that the isometric twitch torque after the conditioning contraction at each measurement time was larger than that before the conditioning contraction (*p* < 0.001). Time-course changes of the isometric twitch torque were identical between the two velocity conditions.

**Figure 4:**
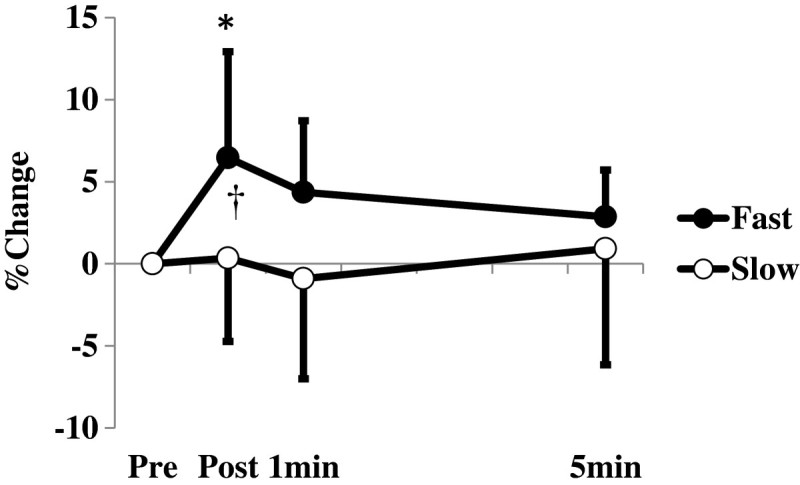
**Relative changes in the maximal voluntary concentric torque.** Relative changes in the maximal voluntary concentric torque after the conditioning contraction. Pre: Before the conditioning contraction; Post: Immediately after the conditioning contraction. *: Significant difference (*p* < 0.05) from Pre value in the fast velocity condition. †: Significant difference (*p* < 0.05) between two conditions.

For the maximal voluntary concentric torque, there was a significant interaction of time point and velocity (*F* value = 3.343, *p* = 0.031) (Figure 4). Post-hoc tests revealed that the maximal voluntary concentric torque after the conditioning contraction in the fast velocity condition was significantly greater than that before the conditioning contraction (*p* = 0.003), whereas that in the slow velocity condition did not change (*p* = 0.705). Moreover, the relative change of the maximal voluntary concentric torque immediately after the conditioning contraction was significantly greater in the fast velocity condition than in the slow velocity condition (*p* = 0.005) (Figure 4).

There was no significant interaction of time point and velocity in the M-wave amplitude of each muscle (*F* value = 0.405 - 0.549, *p* = 0.652 - 1.000), but the main effect of time was observed only in the SOL (*F* value = 4.560, *p* = 0.009). The M-wave amplitude of the SOL immediately after the conditioning contraction was larger than that before the conditioning contraction (*p* < 0.001) (Table 2). The interaction of time and velocity (*F* value = 0.013 - 0.740, *p* = 0.536 - 0.998) and the main effects of the two factors (*F* value = 0.015 - 2.548, *p* = 0.073 - 0.906) were not significant in the RMS_EMG_ of each muscle (Table 3). For the values of RMS_EMG_ normalized by the M-wave, no significant interaction was found in all muscles (*F* value = 0.333 - 0.677, *p* = 0.573 – 0.804), but significant main effects were found between two velocity conditions in LG (*F* value = 10.461, *p* = 0.008) and SOL (*F* value = 4.796, *p* = 0.007), and among time points in SOL (*F* value = 4.560, *p* = 0.009) (Table 4). Additional analyses revealed that the value of RMS_EMG_ normalized by the M-wave in SOL immediately after the conditioning contraction was significantly smaller than that before the conditioning contraction (*p* = 0.001).

**Table 2:** **Relative changes of M-wave amplitude after the conditioning contraction**

M wave (%Change relative to Pre values)			
**MG**	**Post**	**1 min**	**5 min**
**Fast**	**-7.4 ± 13.4**	**4.4 ± 9.1**	**8.4 ± 7.5**
**Slow**	**-3.7 ± 16.6**	**0.7 ± 9.7**	**6.3 ± 7.4**
**LG**	**Post**	**1 min**	**5 min**
**Fast**	**4.6 ± 11.9**	**5.6 ± 6.5**	**5.2 ± 6.7**
**Slow**	**2.9 ± 4.2**	**7.3 ± 8.9**	**5.5 ± 12.0**
**SOL**	**Post**	**1 min**	**5 min**
**Fast**	12.8 ± 14.6*	**3.3 ± 12.4**	**1.2 ± 9.2**
**Slow**	10.9 ± 14.6*	**3.6 ± 7.0**	**1.1 ± 6.1**

Data are shown as means ± SDs. *Significant difference (*p* < 0.05) from Pre value. Pre: Before the conditioning contraction; Post: Immediately after the conditioning contraction; MG: Medial gastrocnemius; LG: Lateral gastrocnemius; SOL: Soleus.

**Table 3:** **Relative changes of RMS**_**EMG**_ **amplitude during maximal voluntary concentric contraction after the conditioning contraction**

RMS_EMG_(%Change relative to Pre values)			
**MG**	**Post**	**1 min**	**5 min**
**Fast**	**-3.4 ± 13.0**	**-7.3 ± 19.2**	**-6.4 ± 14.8**
**Slow**	**-4.6 ± 14.7**	**-7.9 ± 22.1**	**-7.9 ± 16.2**
**LG**	**Post**	**1 min**	**5 min**
**Fast**	**-7.5 ± 15.1**	**-13.9 ± 16.6**	**-8.5 ± 14.3**
**Slow**	**-2.4 ± 19.0**	**-11.9 ± 17.9**	**-9.3 ± 22.7**
**SOL**	**Post**	**1 min**	**5 min**
**Fast**	**-2.4 ± 18.5**	**-1.8 ± 21.4**	**-2.8 ± 18.6**
**Slow**	**-9.9 ± 4.8**	**-9.6 ± 13.1**	**-6.1 ± 6.7**
**TA**	**Post**	**1 min**	**5 min**
**Fast**	**2.4 ± 15.3**	**-5.1 ± 17.3**	**-0.1 ± 19.5**
**Slow**	**-3.2 ± 13.7**	**-11.7 ± 7.5**	**-1.8 ± 12.3**

Data are shown as means ± SDs. Pre: Before the conditioning contraction; Post: Immediately after the conditioning contraction; MG: Medial gastrocnemius; LG: Lateral gastrocnemius; SOL: Soleus; TA: Tibialis anterior.

**Table 4:** **The values of RMS**_**EMG**_ **normalized by the M-wave**

RMS_EMG_normalized by M-wave						
MG		Pre	Post		1 min	5 min
Fast		0.118 ± 0.106	0.124 ± 0.108		0.115 ± 0.125	0.101 ± 0.095
Slow		0.106 ± 0.080	0.108 ± 0.091		0.099 ± 0.085	0.092 ± 0.082
LG		Pre	Post		1 min	5 min
Fast	#	0.074 ± 0.037	0.064 ± 0.027		0.062 ± 0.037	0.065 ± 0.032
Slow		0.063 ± 0.033	0.058 ± 0.023		0.052 ± 0.027	0.057 ± 0.036
SOL		Pre	Post		1 min	5 min
Fast	#	0.054 ± 0.028	0.047 ± 0.026	*	0.052 ± 0.033	0.052 ± 0.024
Slow		0.047 ± 0.022	0.038 ± 0.015		0.042 ± 0.020	0.044 ± 0.021

Data are shown as means ± SDs. *Significant difference (*p* < 0.05) from Pre value. #Significant difference (*p* < 0.05) between two velocity conditions. Pre: Before the conditioning contraction; Post: Immediately after the conditioning contraction; MG: Medial gastrocnemius; LG: Lateral gastrocnemius; SOL: Soleus.

For the ankle joint angle at peak torque during the maximal voluntary concentric contraction occurred, there were no significant interaction (*F* value = 0.39, *p* = 0.762) and main effects (velocity; *F* value = 0.34, *p* = 0.572, time point; *F* value = 2.114, *p* = 0.117) (Table 5).

**Table 5:** **Ankle joint angle at peak torque during the maximal voluntary concentric contraction occurred**

Ankle joint angle (degree)				
	**Pre**	**Post**	**1 min**	**5 min**
**Fast**	**-8.7 ± 1.6**	**-8.8 ± 2.2**	**-9.3 ± 1.5**	**-8.6 ± 2.1**
**Slow**	**-8.2 ± 2.6**	**-8.9 ± 2.1**	**-9.2 ± 1.4**	**-8.5 ± 2.4**

Data are shown as means ± SDs. Pre: Before the conditioning contraction; Post: Immediately after the conditioning contraction.

**Figure 5:**
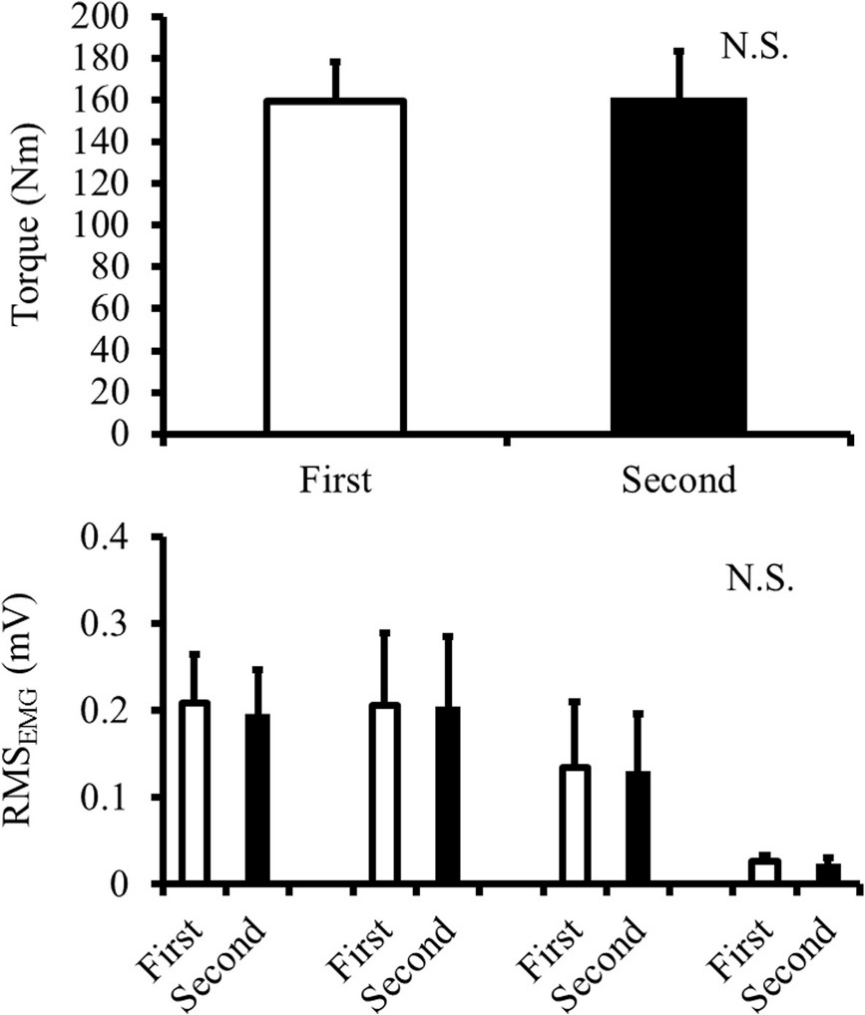
**Mean value of torque and RMS**_**EMG**_**of plantar flexors during the conditioning contraction in first and second conditions.** MG: medial gastrocnemius, LG: lateral gastrocnemius, SOL: soleus, TA: tibialis anterior.

For the mean value of torque and RMS_EMG_ of plantar flexors during conditioning contraction, significant differences were not found (*p* = 0.089 – 0.804) (Figure 5).

## Discussion

There was an increase in the maximal voluntary concentric torque after a conditioning contraction in a fast but not in a slow velocity conditions. This result supports our hypothesis and indicates that the extent of increase in the maximal voluntary concentric torque at the given conditioning contraction is affected by the joint angular velocity during the maximal voluntary concentric contraction.

The isometric twitch torque elicited before each maximal voluntary concentric contraction significantly increased to a similar extent in both velocity conditions. In addition, the time-course changes in isometric twitch torque were similar between the two velocity conditions. In our study, the change in isometric twitch torque was measured to represent the extent of the positive effect of the conditioning contraction. Since twitch contractions were elicited in an identical manner (isometrically in both velocity conditions), the current results indicate that the extent of the positive effect of the conditioning contraction was not different between the two velocity conditions. Thus, the observed difference in the change of maximal voluntary concentric torque between the two velocity conditions should be due to the difference of the sensitivity to the positive effect induced by the conditioning contraction, that is, myosin regulatory light chain phosphorylation (Sweeney et al. 1993).

The extent of increase in muscle force induced by a conditioning contraction becomes smaller as the contraction intensity increases (MacIntosh and Willis 2000). In fact, the force attained during a high-intensity, electrically-evoked isometric contraction was not increased by a conditioning contraction (Vandenboom et al. 1993;1995;1997). The positive effect of a conditioning contraction is prominent when the interaction between actin and myosin is insufficient (Sweeney et al. 1993). The number of attached cross bridges is decreased as the shortening velocity of muscle fibers are increased (Piazzesi et al. 2007). These studies suggest that the positive effect is pronounced when the joint angular velocity is sufficiently high, like in the fast velocity condition in the present study, even though the contraction is performed with the maximal voluntarily effort. On the other hand, it might be assumed that the aforementioned factors (i.e., contraction intensity and shortening velocity) could have depressed the increase in the maximal voluntary concentric torque in the slow velocity condition.

Our result on the shortening velocity-related potentiation was in line with previous study examining the influence of a conditioning contraction on the subsequent electrically-evoked high-intensity contractions with different shortening velocities (Gittings et al. 2012). However, because we adopted voluntary instead of electrically-evoked contraction that was conducted before and after the conditioning contraction, the influence of the ways the muscle was excited on the magnitude of potentiation effect should be mentioned. De Haan (1998) reported that in the isolated muscle fibers, the minimum stimulation frequency required for inducing the maximal-intensity contraction was very high, especially when the shortening velocity was high. On the other hand, the typical firing frequency during the maximal voluntary concentric contraction (Harwood et al. 2011) is lower than the minimum stimulation frequency required to electrically evoke the maximal-intensity contraction at high shortening velocity. Thus, the firing frequency during the maximal voluntary concentric contraction, especially in the fast condition, may be insufficient to induce the adequate Ca^2+^ release to evoke the maximal-intensity contraction. If Ca^2+^ is not supplied sufficiently to evoke the maximal-intensity contraction, the increase in twitch force induced by the conditioning contraction is large (MacIntosh and Willis 2000). Therefore, the velocity-related potentiation may be more prominent in the maximal voluntary concentric contraction than electrically-evoked high-intensity concentric contraction due to the insufficient firing frequency in the fast joint angular velocity condition.

The increase in the maximal voluntary concentric torque was found only immediately after the conditioning contraction although the increase in isometric twitch torque was observed five minutes after conditioning contraction. This difference can be explained by sensitivity to the potentiation effect of a conditioning contraction (i.e., myosin regulatory light chain phosphorylation which is considered to be the primary mechanism of PAP) (Sweeney et al. 1993). Ca^2+^ concentration is much higher in the maximal voluntary concentric contraction (multiple impulses) than in twitch contraction (single impulse). In addition, the extent of increase in torque by conditioning contraction becomes smaller when the Ca^2+^ concentration is higher (Persechini et al. 1985). Thus, the increasing effect of the maximal voluntary concentric torque would have diminished faster than that in the isometric twitch torque.

In addition, the joint angle during the maximal voluntary concentric contraction and M-wave amplitude can be influential factors on the increase in torque induced by conditioning contraction. For the first aspect, the joint angle at the instance of torque measurement during a contraction following a conditioning contraction affects the extent of PAP (Vandervoort et al. 1983). In this study, the joint angle at peak torque during the maximal voluntary concentric contraction occurred was not different between the two velocity conditions. Thus, this factor should not affect the difference in the change of the maximal voluntary concentric torque between the two velocity conditions. For the second aspect, the amplitude of M-wave of the SOL increased after the conditioning contraction in both velocity conditions, which can affect the muscle force. In addition, the values of RMS_EMG_ normalized by the M-wave were not constant among time points. However, the current result that the time course changes in M-wave, RMS_EMG_ and RMS_EMG_ normalized by the M-wave were similar (no significant interaction) between the two velocity conditions discards the possibility that the observed velocity-related difference in the magnitude of increase in the maximal voluntary concentric torque is due to the influence of central or peripheral nervous system.

In our study, we found that the maximal voluntary concentric torque obtained during slow velocity condition was not increased by the conditioning contraction. However, it was practically difficult to find the threshold of joint angular velocity above which the maximal voluntary concentric torque was increased. This is because the extent of increase in the maximal voluntary concentric torque was not large (about 7% in fast velocity condition) with respect to the coefficient of variance of the measurement for maximal voluntary concentric torque (about 5% in our experiment). Thus, it was difficult to identify the point at which the positive effect emerged. Further studies adopting electrically-evoked tetanic concentric contraction (higher reproducibility than voluntary contraction) may be able to delineate the velocity effect.

## Conclusion

The extent of increase in the maximal voluntary concentric torque induced by the conditioning contraction is influenced by the joint angular velocity. The maximal voluntary concentric torque can be potentiated by the conditioning contraction when the joint angular velocity during the maximal voluntary concentric contraction is sufficiently high, and not when the velocity is critically slow.

## Consent

Written informed consent was obtained from the patient for the publication of this report and any accompanying images.

## Authors’ original submitted files for images

Below are the links to the authors’ original submitted files for images. Authors’ original file for figure 1 Authors’ original file for figure 2 Authors’ original file for figure 3 Authors’ original file for figure 4 Authors’ original file for figure 5
