# Supplementary material for: Potentiation of isokinetic torque is velocity-dependent following an isometric conditioning contraction
Source: Springerplus. 2013 Oct 22;2(1):554. doi: 10.1186/2193-1801-2-554 (PMC3825088; doi:10.1186/2193-1801-2-554)
Supplement: Supplementary file 1 — Authors’ original file for figure 1 [file 40064_2013_613_MOESM1_ESM.pdf]

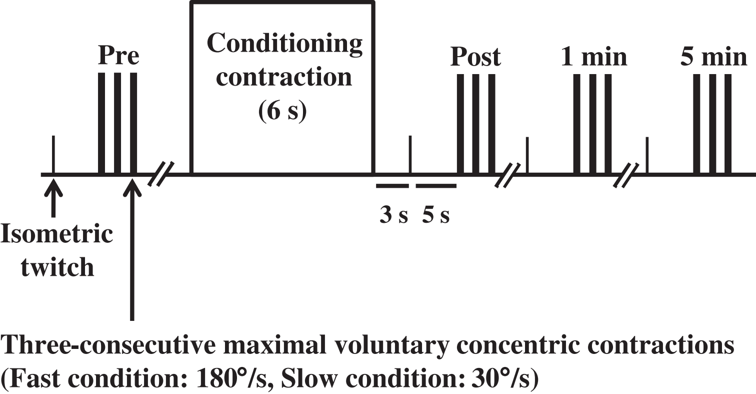
Pre
Conditioning
contraction
(6 s)
Post
1 min
5 min
3 s
5 s
Isometric
twitch
Three-consecutive maximal voluntary concentric contractions
(Fast condition: 180°/s, Slow condition: 30°/s)
