# Supplementary figures and images for: Potentiation of isokinetic torque is velocity-dependent following an isometric conditioning contraction
Source: Springerplus. 2013 Oct 22;2(1):554. doi: 10.1186/2193-1801-2-554 (PMC3825088; doi:10.1186/2193-1801-2-554)

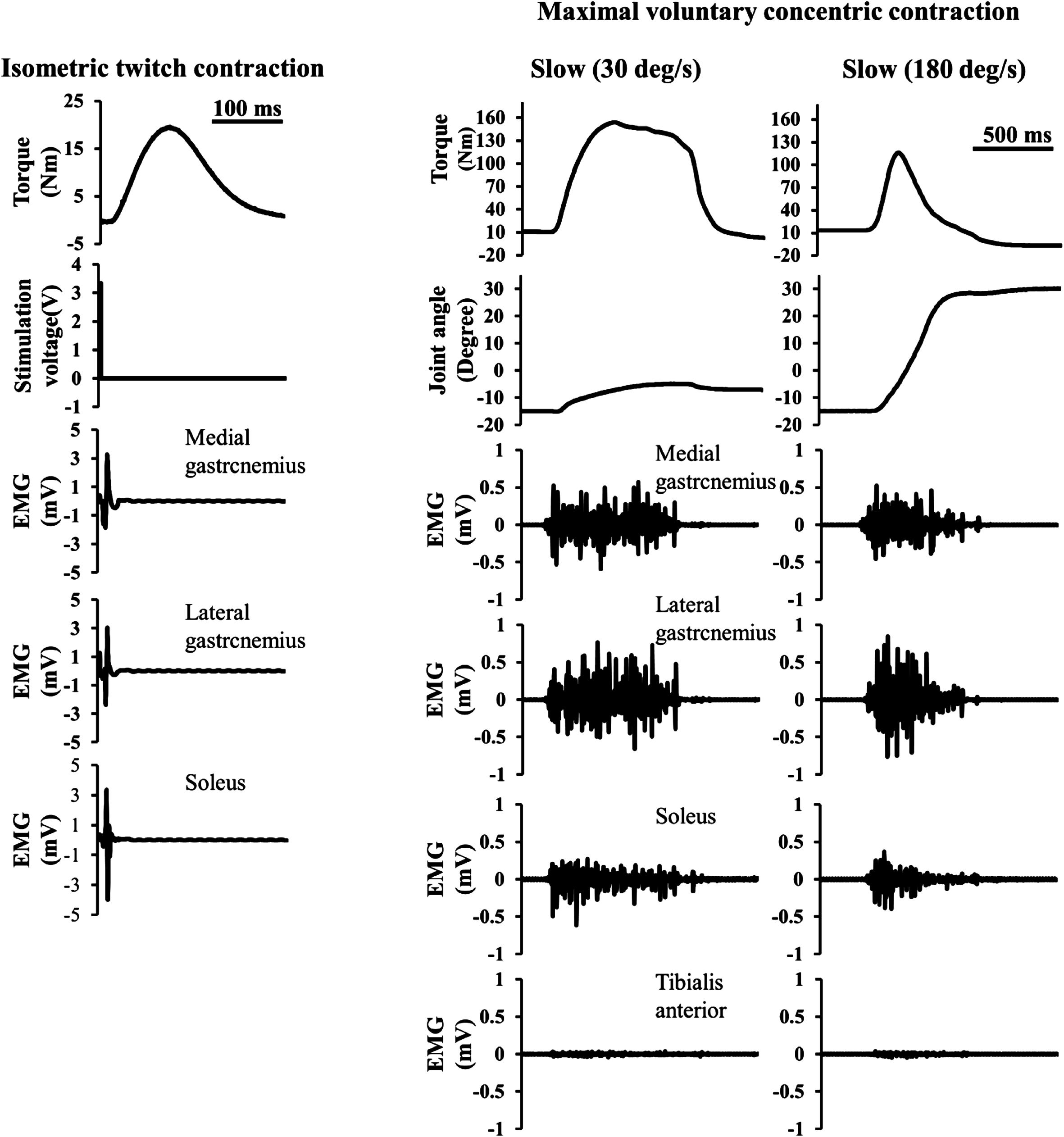

Supplement: Supplementary file 2 — Authors’ original file for figure 2 [file 40064_2013_613_MOESM2_ESM.tiff]

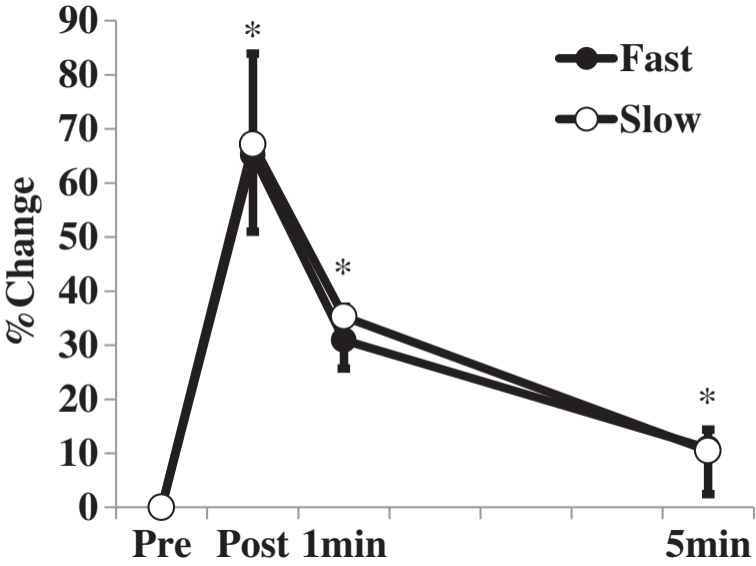

Supplement: Supplementary file 3 — Authors’ original file for figure 3 [file 40064_2013_613_MOESM3_ESM.pdf]

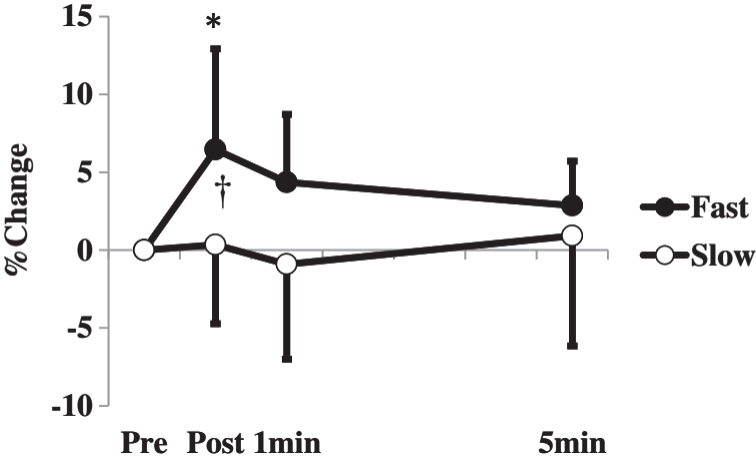

Supplement: Supplementary file 4 — Authors’ original file for figure 4 [file 40064_2013_613_MOESM4_ESM.pdf]

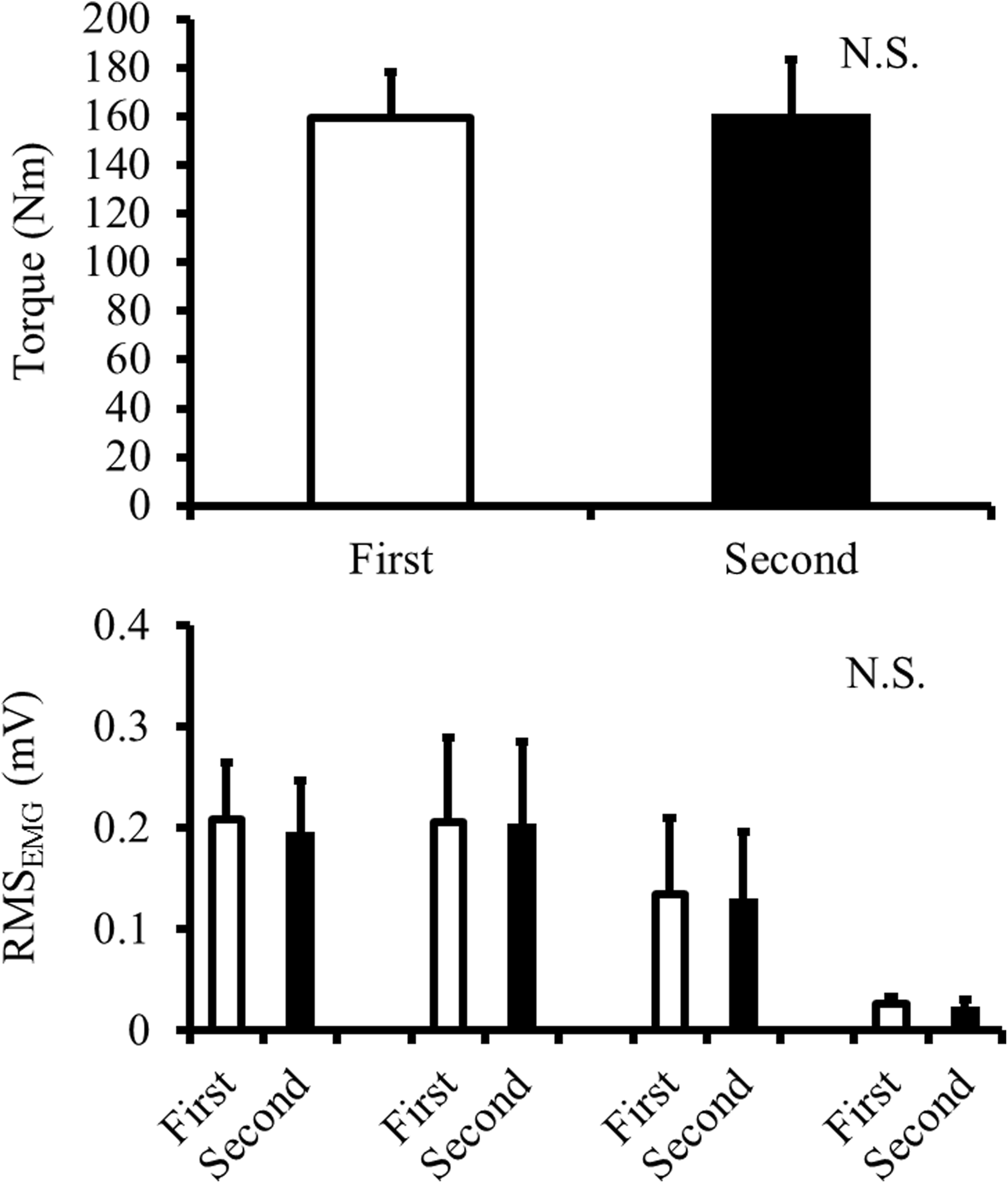

Supplement: Supplementary file 5 — Authors’ original file for figure 5 [file 40064_2013_613_MOESM5_ESM.tif]
